# Pan-Cancer Analysis of FURIN as a Potential Prognostic and Immunological Biomarker

**DOI:** 10.3389/fmolb.2021.648402

**Published:** 2021-04-22

**Authors:** Bolun Zhou, Shugeng Gao

**Affiliations:** Thoracic Surgery Department, National Cancer Center, National Clinical Research Center for Cancer, Cancer Hospital, Chinese Academy of Medical Sciences and Peking Union Medical College, Beijing, China

**Keywords:** pan-cancer, FURIN, tumor immunity, prognosis, biomarker

## Abstract

**Background:**

Furin is a calcium-dependent protease that processes various precursor proteins through diverse secretory pathways. The deregulation of FURIN correlated with the prognosis of patients in numerous diseases. However, the role of FURIN in human pan-cancer is still largely unknown.

**Methods:**

Multiple bioinformatic methods were employed to comprehensively analyze the correlation of FURIN expression with prognosis, mismatch repair (MMR), microsatellite instability (MSI), tumor mutational burden (TMB), DNA methylation, tumor immune infiltration, and common immune checkpoint inhibitors (ICIs) from the public database, and aim to evaluate the potential prognostic value of FURIN across cancers.

**Results:**

FURIN was aberrantly expressed and was strongly correlated with MMR, MSI, TMB, and DNA methylation in multiple types of cancer. Moreover, survival analysis across cancers revealed that FURIN expression was correlated with overall survival (OS) in four cancers, disease-specific survival (DSS) in five cancers, progression-free interval (PFI) in seven cancers, and disease-free interval (DFI) in two cancers. Also, FURIN expression was related to immune cell infiltration in 6 cancers and ImmuneScore/StromalScore in 10 cancers, respectively. In addition, FURIN expression also showed strong association between expression levels and immune checkpoint markers in three cancers.

**Conclusion:**

FURIN can serve as a significant prognostic biomarker and correlate with tumor immunity in human pan-cancer.

## Introduction

Furin, proprotein convertase subtilisin/kexin family member 3, is a calcium-dependent protease that is ubiquitously expressed in vertebrates (Seidah et al., 1998). A 794-amino acid zymogen, expressed by FURIN, undergoes two steps of autocatalytic cleavage before its full activation (Anderson et al., 1997). Furin belongs to proprotein convertases (PCs) that function in converting proproteins into bioactive proteins and peptides through limited proteolysis (Jaaks and Bernasconi, 2017). Furin and other PCs process a large number of precursor proteins, such as hormones, neuropeptides, growth factors and their receptors, bacterial toxins, and adhesion molecule, through various secretory pathways (Seidah and Prat, 2012). Thus, the deregulation of FURIN is correlated with a variety of pathological conditions, including cardiovascular disease (Yakala et al., 2019), rheumatoid disease (Valli et al., 2019), endocrinopathies (Al Rifai et al., 2017), and central nervous system disorder (Yang et al., 2018). As for cancers, several studies have found that FURIN is expressed in various cancers and sarcomas (Khatib et al., 2002; Coppola et al., 2008; Lee et al., 2016; Jaaks and Bernasconi, 2017). For instance, genetic silencing of FURIN inhibits processing of the insulin-like growth factor 1 receptor (IGF1R), migration, and invasion in lung cancers, which indicates that furin is a potential therapeutic target in lung cancers (Mbikay et al., 1997; Bassi et al., 2017). Furin also promotes the process of IGF1, migration, and invasion in rhabdomyosarcoma (Jaaks et al., 2016). Also, it has been reported that the downregulation of FURIN resulted in the inhibition of complete matrix metalloprotease-2 (MMP-2) maturation and invasiveness of fibrosarcoma. Although the role of furin in cancers, such as lung cancers and rhabdomyosarcoma, has been studied, its roles across the human cancer spectrum still remain largely unknown.

The tumor microenvironment (TME) contains complex interactions between tumors and the microenvironment, which is a trending focus of cancer research. One of the basic TME components is immune cell infiltration. TME contains different kinds of immune cells, including neutrophils, macrophages, natural killer cells, and dendritic cells. Immune cells in TME have been monitoring cancer cells throughout their life time to prevent cancer cells from proliferating and growing (Zitvogel et al., 2013). However, cancer can progress if immune cells fail to eliminate the preneoplastic cells in time. Cancer cells use a special strategy to escape from immune-mediated killing in order to survive (Gajewski et al., 2013; Quail and Joyce, 2013). Besides, some inflammatory cells, such as neutrophils, block the function of the immune cells of TME and promote the growth of cancer cells (Kumar et al., 2016; Powell and Huttenlocher, 2016). On the contrary, tumor-associated macrophages (TAMs), one of the tumor-infiltrating lymphocytes, regulate immune escape and also serve as an essential factor in tumor progression, which can affect the efficacy of immunotherapy (Ovais et al., 2019; Zhu et al., 2019). At present, more potential targets for immunotherapy have been discovered. For example, previous studies have proved that PD-1/PD-L1 inhibitors play an essential antitumor role in lung cancer and melanoma by blocking its signaling pathway of apoptosis (Topalian et al., 2015; Shen and Zhao, 2018; Cordonnier et al., 2020; Schoenfeld et al., 2020). However, immunotherapies are not fully used in cancers and many patients do not respond well to immunotherapies. Therefore, it is urgent to identify more specific immune biomarkers and discover the relationship between cancer and immunology, which can be used to treat patients.

In the present study, we systematically investigated 33 types of cancer to find out the correlation of FURIN expression with the prognosis of patients. Furthermore, this study totally explored immune checkpoints and 6 tumor-infiltrating immune cells in the TME of 33 cancers to find the correlation of FURIN expression with them. The findings of this study revealed that furin affects the prognosis of patients and interacts with infiltrating immune cells in many cancers, specifically in brain lower grade glioma (LGG).

## Materials and Methods

### Analysis of FURIN Expression and Sample Data Across Multiple Cancers

FURIN expression information in 31 types of normal tissues (e.g., brain, breast, colon, and kidney) was downloaded through the Genotype-Tissue Expression (GTEx) portal^1^ and 21 tumor cell lines (e.g., lung, liver, kidney, and intestine) were downloaded *via* the Cancer Cell Line Encyclopedia (CCLE) database^2^. FURIN expression data was obtained from the GTEx database for normal tissues and The Cancer Genome Atlas (TCGA) database for cancer tissues, which were combined to evaluate FURIN expression in cancer tissues compared with it in normal tissues. Clinical follow-up information and level 3 RNA sequencing data for patients of 33 cancers were obtained from the TCGA database. Log2 conversion was performed to normalize the expression data. R software 3.6.2 was applied to perform the statistical analysis.

### Analysis of MMR Gene Mutation and DNA Methylation

DNA mismatch repair (MMR) is able to rectify DNA replication errors and attenuate chromosomal rearrangements, which is also related to tumorigenesis (Iyer et al., 2015; Baretti and Le, 2018). MLH1, MSH2, MSH6, EPCAM, and PMS2 are five MMR genes, and their expression levels in multiple cancers were obtained from the TCGA database. The correlation of expression levels of MMR genes with the expression levels of FURIN was analyzed by the Spearman’s correlation method. In addition, DNA methylation serves as a necessary factor in affecting gene expression. In the present study, we evaluated the expression levels of DNMT1, DNMT2, DNMT3A, and DNMT3B, and used the Spearman’s correlation method to evaluate the correlation of these four methyltransferases with FURIN expression.

### Analysis of Survival and Prognosis

The clinical information of different cancers was obtained from the TCGA database. The correlation of FURIN expression with the prognosis of patients [OS, disease-specific survival (DSS), progression-free interval (PFI), and disease-free interval (DFI)] in 33 kinds of cancers was revealed by the Kaplan–Meier curves. The univariate survival analysis was applied to calculate the *p*-value.

### Correlation Between FURIN Expression and Tumor Mutational Burden or Microsatellite Instability Across Cancers

Tumor mutational burden (TMB) refers to total mutations in the DNA carried by tumor cells (Chalmers et al., 2017). The “maftools” R package was applied to analyze the somatic data (MAF data) in human pan-cancer from the TCGA database. We then calculated the number of mutations of exons to identify the TMB in each cancer. Microsatellite instability (MSI) refers to the simultaneous gain or loss of nucleotides of the repetitive DNA fragments (Li et al., 2020). We obtained the MSI score from the TCGA database. The analysis of the association between FURIN expression and TMB or MSI was based on the Spearman’s method.

### Correlations of FURIN Expression With Immune Characteristics

We systematically analyzed the infiltration levels of B cells, CD4(+) T cells, CD8(+) T cells, macrophages, dendritic cells, and neutrophils among different types of cancers. The infiltrating immune cell scores of these cells in 33 cancers were obtained from the Tumor Immune Evaluation Resource (TIMER) database^3^. The correlation of FURIN expression with the immune infiltrating scores of these six immune cells was evaluated by the Spearman’s correlation analysis. Furthermore, we obtained the ImmuneScore and StromalScore of multiple cancers *via* the “estimate” R package. We then used Spearman’s correlation method to analyze the correlation of FURIN expression with ImmuneScore and StromalScore. In addition, we used the Spearman’s correlation analysis to evaluate the relationships between FURIN expression and expression levels of the immune checkpoint markers. ALL of the gene expression levels were log2 transformed.

### Statistical Analysis

The Kruskal–Wallis test was applied to evaluate FURIN expression in normal and cancer tissues. The Wilcoxon test was applied to evaluate the differences of the expression levels FURIN between tumor and normal tissues. In survival analysis, the relationship between FURIN expression and the survival of patients was evaluated by the univariate Cox regression. The *p*-value was also calculated by the univariate Cox regression. The Kaplan–Meier method was applied to analyze the prognosis of patients in accordance with the diverse expression levels of FURIN. The Spearman’s correlation analysis was used to evaluate the correlation of FURIN expression with methyltransferase levels, expression levels of MMR gene, and expression of immune checkpoint genes. *R* > 0.20 was considered as positive in correlations and *p* < 0.05 was regarded as the statistical criteria to set thresholds.

## Results

### FURIN Expression Level Is Abnormal in Human Pan-Cancer

**FIGURE 1:**
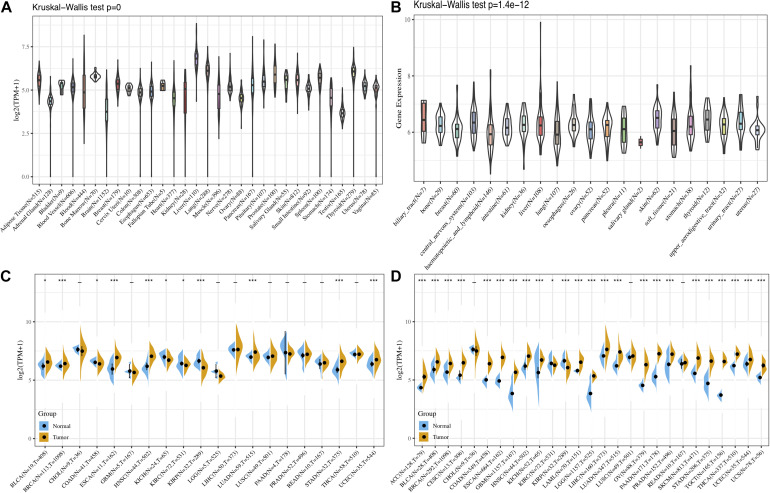
The expression levels of FURIN across cancers. **(A)** Data of the GTEx database showed the expression levels of FURIN in diverse normal tissues. **(B)** Data of the CCLE database showed the expression levels of FURIN in diverse cancer cells. **(C)** Data of the TCGA database showed expression of FURIN in diverse cancers and normal tissues. **(D)** Data of the TCGA database and the GTEx database showed FURIN expression in diverse kinds of tumors (*p < 0.05, **p < 0.01, ***p < 0.001).

First, the GTEx database was applied to evaluate FURIN expression in 31 normal tissues. We found that the expression levels of FURIN in multiple normal tissues were relatively high in the liver, lung, and thyroid tissues, while those in the testis, brain, adrenal gland, and other tissues were relatively low (Figure 1A). Furthermore, we analyzed the expression levels of FURIN in 21 tumor cells based on the data of the CCLE database. FURIN was differentially expressed in different types of tumor cell lines (Figure 1B). In addition, the TCGA database was applied to evaluate FURIN expression in 20 types of tumors and normal tissues, so as to evaluate the difference of FURIN mRNA levels between normal and tumor tissues. Compared with FURIN expression in normal tissues, its expression levels were relatively higher in bladder urothelial carcinoma (BRCA), esophageal carcinoma (ESCA), head and neck squamous cell carcinoma (HNSC), lung adenocarcinoma (LUAD), stomach adenocarcinoma (STAD), and uterine corpus endometrial carcinoma (UCEC). The expression levels of FURIN were relatively lower in KIRC and KIRP than those in normal tissues (Figure 1C). Since the data of TCGA normal samples are far less than that of tumor samples in some types of cancer, we integrated the data of the TCGA database and the GTEx database to evaluate the differences in FURIN expression in different cancers. The results revealed that FURIN was highly expressed in 21 types of cancer, except cholangiocarcinoma (CHOL), lung squamous cell carcinoma (LUSC), and rectum adenocarcinoma (READ) (Figure 1D). The above results showed that FURIN expressed abnormally across multiple cancers.

### FURIN Is Associated With Expression Levels of MMR Gene and DNA Methylation in Human Pan-Cancer

**FIGURE 2:**
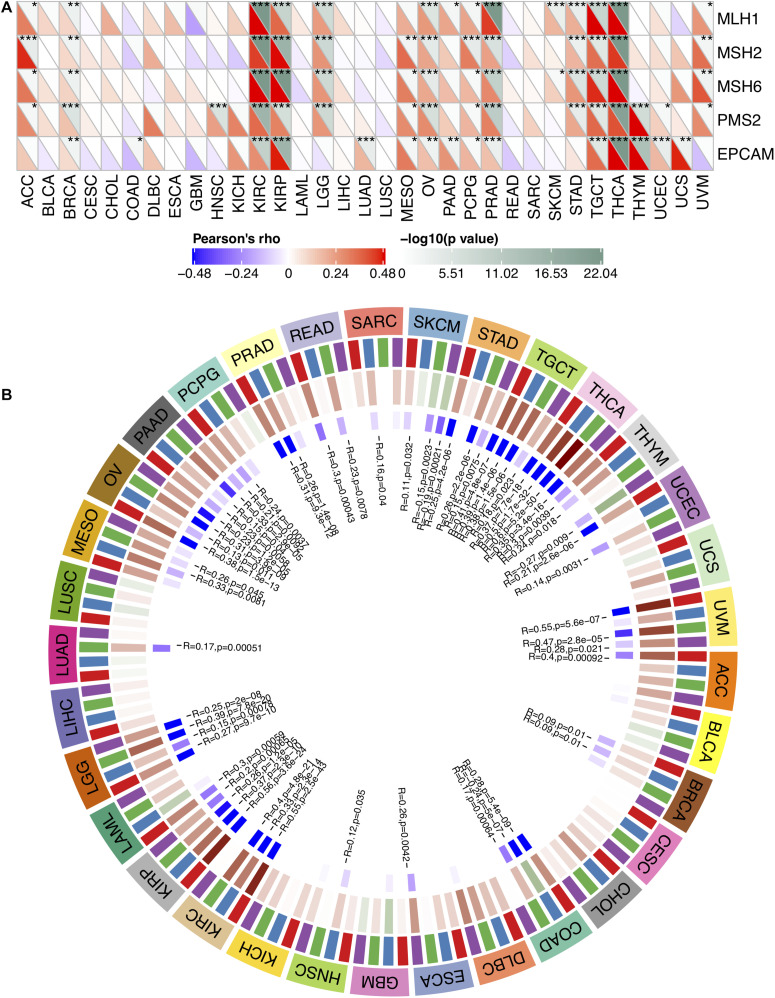
In human pan-cancer, the expression levels of FURIN correlate with expression levels of five MMR genes and four DNA methyltransferases. **(A)** The Spearman’s correlation analysis of FURIN expression with expression levels of five MMR genes across cancers (*p < 0.05, **p < 0.01, ***p < 0.001). **(B)** The Spearman’s correlation analysis of FURIN expression with four DNA methyltransferases across cancers.

MMR serves as a vital factor in maintaining the stability of the genome. It serves specifically for insertion/deletion of mispairs and base–base mismatches during the replication and recombination of DNA (Li, 2008). The deletion of some important genes that play a role in MMR leads to DNA replication and recombination errors, which can cause tumorigenesis (Baretti and Le, 2018). In order to determine the role of FURIN in tumor progression, we evaluated the association of the expression level of FURIN with mutation levels of five MMR genes. As results shown in Figure 2A, FURIN expression was highly related to MMR genes in 14 cancers, including adrenocortical carcinoma (ACC), BRCA, kidney renal clear cell carcinoma (KIRC), kidney renal papillary cell carcinoma (KIRP), brain LGG, mesothelioma (MESO), ovarian serous cystadenocarcinoma (OV), pheochromocytoma and paraganglioma (PCPG), prostate adenocarcinoma (PRAD), STAD, tenosynovial giant cell tumor (TGCT), thymoma (THYM), thyroid carcinoma (THCA), and uveal melanoma (UVM).

DNA methylation is abnormal in multiple cancers and serves as a vital factor in cancer progression (Klutstein et al., 2016; Koch et al., 2018). Therefore, the relationships between FURIN and four DNA methyltransferases were evaluated. As for results shown in Figure 2B, FURIN expression was highly associated with these four DNA methyltransferases in multiple cancers, specifically in colon adenocarcinoma (COAD), KIRP, KIRC, OV, LGG, pancreatic adenocarcinoma (PAAD), STAD, THCA, TGCT, and UVM. To sum up, this study results indicate that FURIN may regulate the tumor progression by mediating repairment of DNA and DNA methylation across cancers.

### Prognostic Potential of FURIN Across Cancers

**FIGURE 3:**
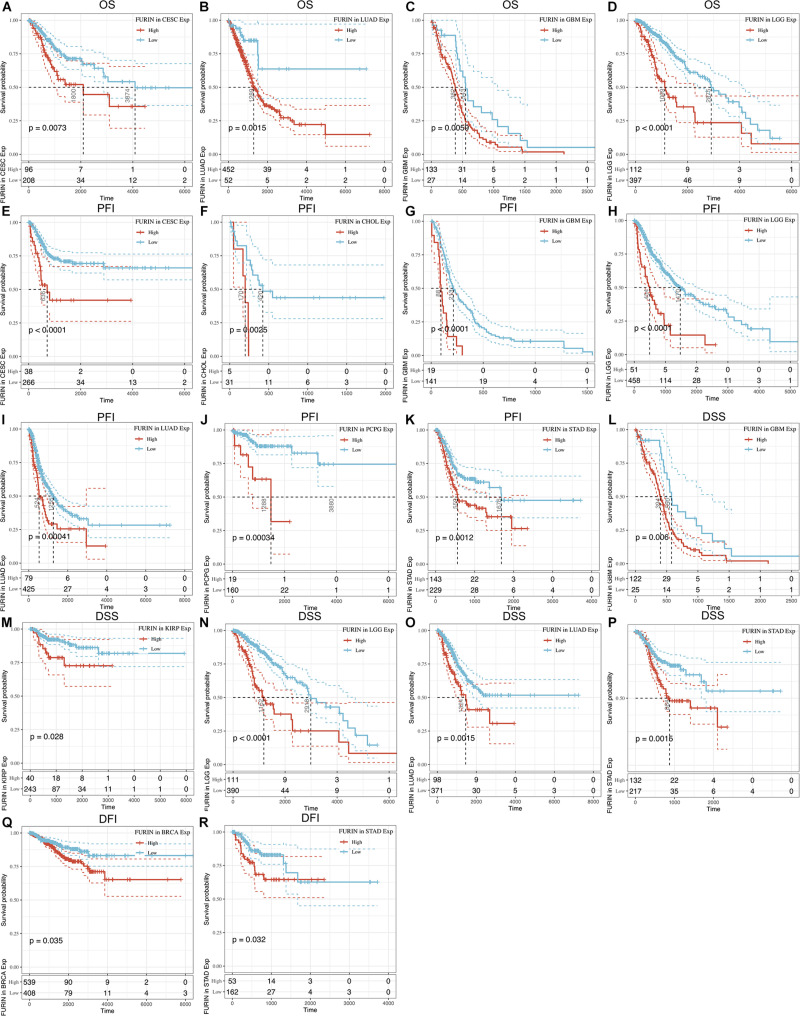
Correlation between FURIN expression in patients with OS, PFI, DSS, and DFI. **(A–D)** Survival analyses of FURIN expression using the Kaplan–Meier OS curves in CESC, LUAD, GBM, and LGG. **(E–K)** Survival analyses of FURIN expression using the Kaplan–Meier PFI curves in CESC, CHOL, GBM, LGG, LUAD, and PCPG. **(L–P)** Survival analyses of FURIN expression using the Kaplan–Meier DSS curves in GBM, KIRP, LGG, LUAD, and STAD. **(Q,R)** Survival analyses of FURIN expression using the Kaplan–Meier DFI curves in BRCA and STAD.

We evaluated the relationship between FURIN and the prognosis of patients across cancers (Table 1). According to this study results, FURIN expression significantly affected the OS of patients in CESC, GBM, LGG, and LUAD across cancers (Figures 3A–D). FURIN acted as a risk factor in cervical squamous cell carcinoma (CESC, *N* = 304, *p* = 0.0073), LUAD (*N* = 504, *p* = 0.0015), glioblastoma multiforme (GBM, *N* = 160, *p* = 0.0059), and LGG (*N* = 509, *p* < 0.0001). Then we found the correlation of FURIN expression with PFI of patients, which indicated that high expression of FURIN unfavorably impacted PFI in CESC (*N* = 304, *p* < 0.0001), CHOL (*N* = 36, *p* = 0.025), GBM (*N* = 160, *p* < 0.0001), LGG (*N* = 509, *p* < 0.0001), LUAD (*N* = 504, *p* = 0.00041), PCPG (*N* = 179, *p* = 0.00034), and STAD (*N* = 372, *p* = 0.012) (Figures 3E–K). Besides, we analyzed the relationship between FURIN expression and the DSS of patients across cancers. FURIN expression was significantly related to the DSS of patients in five cancers (GBM, KIRP, LGG, LUAD, and STAD) (Figures 3L–P). High FURIN expression was closely related to unfavorable prognosis in GBM (*N* = 147, *p* = 0.006), KIRP (*N* = 283, *p* = 0.028), LGG (*N* = 501, *p* < 0.0001), LUAD (*N* = 469, *p* = 0.0015), and STAD (*N* = 349, *p* = 0.0015) *via* the Kaplan–Meier method. Moreover, we assessed the relationship between FURIN expression and DFI. The Kaplan–Meier method revealed that FURIN expression correlated with DFI in two types of cancers, namely BRCA and STAD. The high expression levels of FURIN negatively impacted DFI in BRCA (*N* = 947, *p* = 0.035) and STAD (*N* = 215, *p* = 0.032) (Figures 3Q–R). To sum up, the above results show that FURIN expression is related to the prognosis of patients significantly, especially in those with LUAD, LGG, and GBM.

**TABLE 1:** Overall information of FURIN in human pan-cancer.

Characteristics	OS	PFI	DSS	DFI	TMB	MSI	TME	ImmuneScore	StromalScore
BLCA								P	
BRCA				N	N				
CESC	N	N				P			
CHOL		N						N	
COAD						P	P	P	
DLBC						N		P	P
HNSC						N			P
LGG	N	N	N				P		P
LIHC									P
LUAD	N	N	N				N	N	
LUSC						P			
GBM	N	N	N		N			P	P
KIRC					N	P	P		P
KIRP			N		N		P		
PAAD						N			
PCPG		N					P	P	P
PRAD					N				
READ						N			P
SARC						P			
SCRC					P				
SKCM						N			
STAD		N	N	N					
TGCT								N	
THCA					N	N			
THYM					P			N	P
UVM								P	P

*N, negatively correlated; P, positively correlated.*

### Correlation Between FURIN Expression and TMB or MSI Across Cancers

**FIGURE 4:**
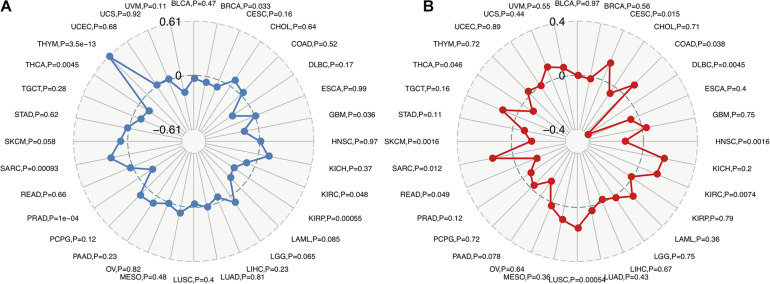
Correlations of FURIN expression with TMB and MSI across cancers. **(A)** The correlation of FURIN expression with TMB across cancers was illustrated by the radar chart. The value in black reveals the range and the curve in blue reveals the correlation coefficient. **(B)** The correlation between FURIN expression and MSI across cancers was illustrated by the radar chart. The value in black reveals the range and the curve in red reveals the correlation coefficient.

Recent studies have shown that TMB may be used as a biomarker and predict the response to immune checkpoint inhibitors (ICIs), including PD-1/PD-L1 (Yarchoan et al., 2017; Chan et al., 2019; Samstein et al., 2019). The analysis of the relationship between FURIN expression and TMB across cancers is meaningful. This study revealed that FURIN expression was positively related to TMB in sporadic colorectal cancer (SCRC) and THYM. On the contrary, FURIN expression was negatively associated with TMB in BRCA, GBM, KIRC, KIRP, PRAD, and THCA (Figure 4A). MSI occurs in multiple cancers and acts as a novel biomarker for ICIs, including PD-1(Dudley et al., 2016; Hause et al., 2016). Thus we then evaluated the correlation of FURIN expression with MSI in human pan-cancer. According to this study results, the FURIN expression was positively related to MSI in CESC, COAD, KIRC, LUSC, and sarcoma (SARC). In contrast, FURIN expression was negatively related to MSI in lymphoid neoplasm diffuse large B-cell lymphoma (DLBC), HNSC, PAAD, READ, skin cutaneous melanoma (SKCM), and THCA (Figure 4B).

### FURIN Expression Is Related to Immune Infiltration Levels and Immune Checkpoint Biomarkers in Human Pan-Cancer

**FIGURE 5:**
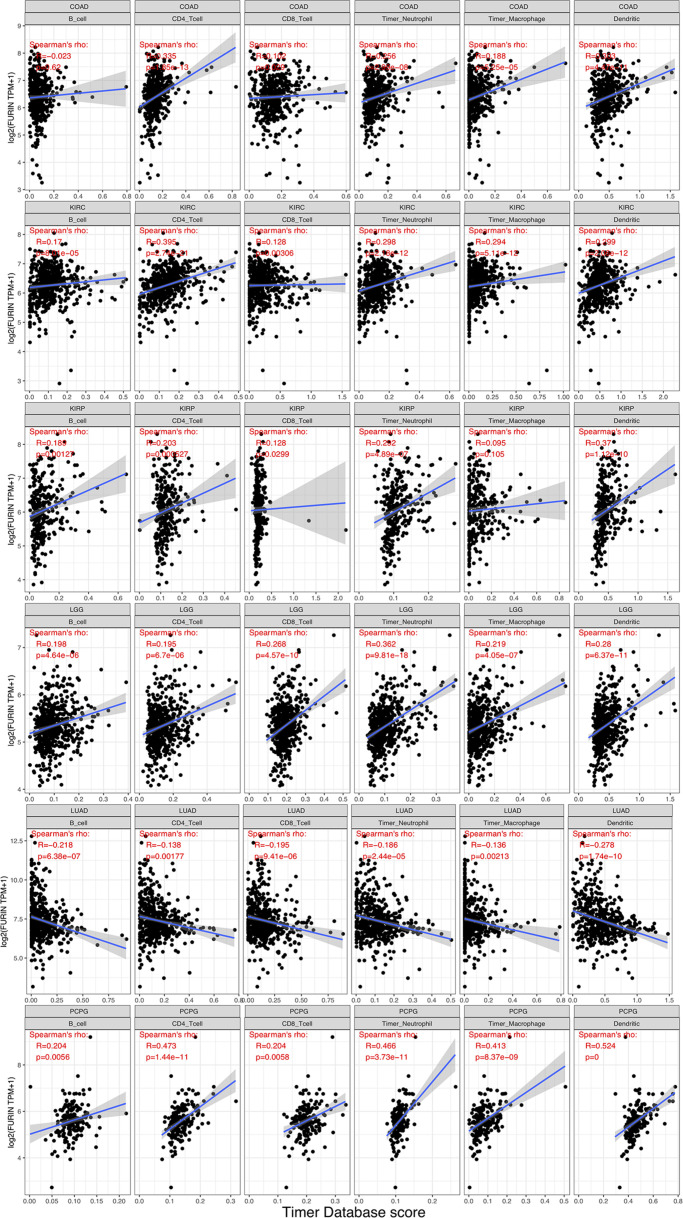
FURIN expression positively correlates with immune infiltration levels in COAD, KIRC, KIRP, LGG, and PCPG. FURIN expression negatively correlates with immune infiltration levels in LUAD.

**FIGURE 6:**
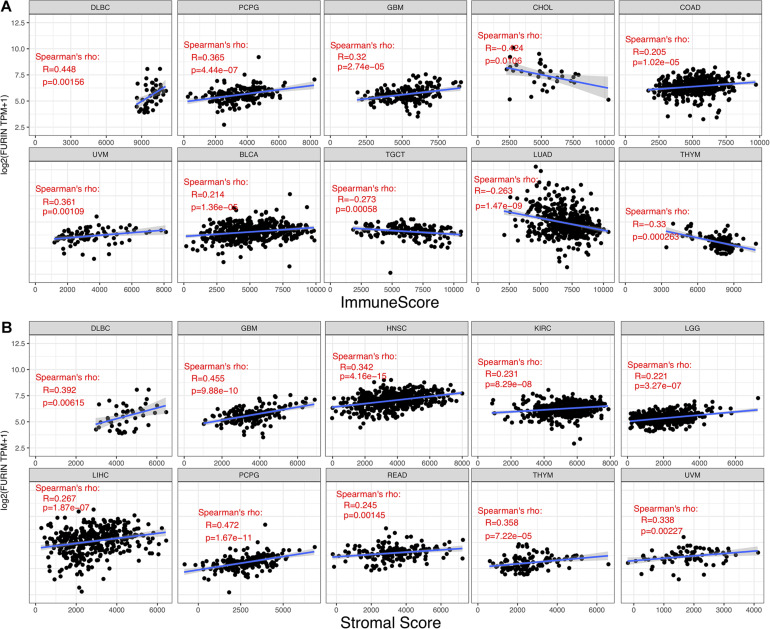
Correlation of FURIN expression with ImmuneScore and StromalScore in various cancers. **(A)** Correlation of FURIN expression with ImmuneScore in DLBC, PCPG, GBM, UVM, BLCA, TGCT, LHAD, and THYM. **(B)** Correlation of FURIN expression with StromalScore in DLBC, GBM, HNSC, KIRC, LGG, LIHC, PCPG, READ, THYM, and UVM.

TME contains a variety of infiltrating immune cells, which can serve as a prognostic biomarker in diverse types of cancers (Bader et al., 2020; Toor et al., 2020). Thus, we evaluated the relationship between FURIN expression and levels of immune infiltration across cancers. We found that FURIN expression was mainly associated with six infiltrating immune cells in COAD, LGG, LUAD, KIRP, KIRC, and PCPG (Figure 5). We then integrated ImmuneScore and StromalScore to further evaluate the relationship between FURIN expression and immune infiltration across cancers. According to the results, FURIN expression was positively correlated with the ImmuneScore in BLCA, COAD, DLBC, GBM, PCPG, and UVM, while FURIN expression negatively correlated with the ImmuneScore in CHOL, LUAD, TGCT, and THYM (Figure 6A). Also, FURIN expression positively correlated with the StromalScore in GBM, KIRC, DLBC, HNSC, LGG, liver hepatocellular carcinoma (LIHC), PCPG, READ, THYM, and UVM (Figure 6B).

**FIGURE 7:**
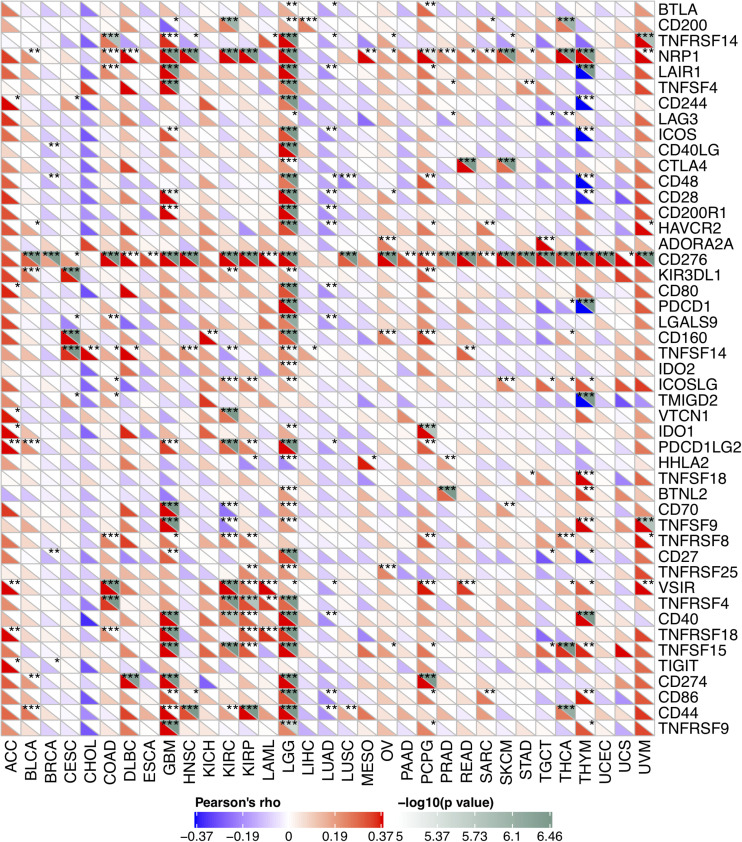
Correlation of FURIN expression with 47 common immune checkpoint gene expression across cancers. *p < 0.05, **p < 0.01, ***p < 0.001.

Due to the association between the expression levels of FURIN and immune infiltration levels, we further studied the association between FURIN and 47 common immune checkpoint genes. Interestingly, according to this study results, FURIN expression correlated with 41 immune checkpoint genes in LGG, 21 immune checkpoint genes in GBM, and 19 immune checkpoint genes in THYM (Figure 7). To conclude, this study results show that FURIN acts significantly in tumor immunity.

## Discussion

The pan-cancer analysis is vital and useful for comparing the similarities and differences among different cancers, which is very helpful to provide a new understanding of cancer prevention and novel tumor biomarkers. Many studies have provided new insights into the whole-genome analysis in human pan-cancer and revealed the close correlation between tumorigenesis and mutations, driver genes, copy number alterations, and tumor purity, which is important for the diagnosis and treatment of various cancers (Zack et al., 2013; Aran et al., 2015; Ma et al., 2018; Saghafinia et al., 2018; Priestley et al., 2019). Furin is a calcium-dependent protease that processes many precursor proteins through various secretory pathways, which is correlated with diverse pathological conditions (Seidah et al., 1998; Seidah and Prat, 2012). Previous studies have evaluated the expression levels and function of furin in several cancers, such as lung cancer (Demidyuk et al., 2013) and HNSC (Bassi et al., 2001). However, the role of furin across cancers and whether it can serve as a prognostic biomarker have not been analyzed, which is clinically meaningful in the studies of tumor. In the present study, we completely evaluated FURIN expression across cancers for the first time. This study results showed that FURIN was aberrantly expressed in 24 cancers and enormously associated with MMR, MSI, DNA methylation, and TMB. Furthermore, the overexpression of FURIN was correlated with a worse prognosis (OS, DSS, PFI, and DFI) in multiple cancers. Besides, FURIN expression was closely associated with the levels of immune infiltration and ICIs in human pan-cancer, specifically in LGG, GBM, and THYM. This study results significantly indicated that FURIN played an essential role in tumor immunity and may act as an important biomarker.

Previous studies have revealed that furin can serve as an important factor in some types of cancer, which suggests that FURIN can be expressed in immune cells and cancer cells. As for the immune cells, Pesu et al. (2008) reported that furin in the T cells can promote to produce more TGF-beta *via* regulatory and effector T cells. Also the production of TGF-beta by T cells triggered by STAT6, which plays an essential role in immune-associated diseases, needed to be processed by furin to be active (Li et al., 2018). In colorectal cancer, furin in cytotoxic T lymphocytes can promote PD-1 expression *via* the nuclear factor kappa-light-chain-enhancer of activated B cells (NF-κB) signaling pathway (Tomé et al., 2019). In glioma, the inhibitor of furin can promote the reactivation of macrophages *via* several antitumor immune factors (Rose et al., 2020). Moreover, FURIN expression was negatively related to the immune infiltration, and inhibition of furin inactivated tumorigenesis and metastasis in triple negative breast cancer (He et al., 2020a). As for cancer cells, Bassi et al. have revealed that furin can increase the invasive potential of head and neck carcinoma *via* combining its substrates such as Transforming growth factor beta (TGF-beta) and Insulin-like growth factor 1 (IGFR-1) (Bassi et al., 2003). Furin can also process Vascular endothelial growth factor C (VEGF-C) to promote angiogenesis, which contributes to the progression of oral tongue squamous cell carcinoma (López de Cicco et al., 2004). In addition, recent studies have shown that furin can activate some tumor-related protein precursors in the KRAS-related ERK-MAPK pathway, thus promoting tumorigenesis of colorectal cancer (He et al., 2020b). In pancreatic cancer, furin contributes to epithelial–mesenchymal transition through the Hippo-YAP pathway and may be used as a therapeutic target (Zhang et al., 2017). In squamous skin cancer, the inhibition of furin in T cells increased immune responses, impaired peripheral immune tolerance, and promoted cancer development (Ortutay et al., 2015; Vähätupa et al., 2016). Furin is also responsible for the progression and metastasis in LUAD and ovarian cancer (Ma et al., 2014; Chen et al., 2020). These studies suggest that furin may serve as a vital factor in different cancers, which may also be related to the prognosis of patients. Compared with previous studies, this study is the first to display the irregular expression landscape of FURIN and analyze the correlation of FURIN expression with the prognosis of patients across cancers. We found that FURIN was aberrantly expressed in multiple cancers and the Kaplan–Meier method revealed that high expression of FURIN was negatively related to the prognosis of patients (OS, DSS, PFI), especially in the case of LGG, GBM, and LUAD. This study results indicate that FURIN can serve as a significant therapeutic target in LGG, GBM, and LUAD. The precise mechanism of FURIN in these cancers is worth exploring in the future.

MMR serves as an important factor in keeping the stability and integrity of the whole genome in normal cells, which mainly contains MSH (MutS homologs) and MLH/PMS (MutL homologs) (Fishel, 2015). Compared with normal tissues, the deficiency of MMR genes in tumor cells leads to higher mutation frequencies and exhibits a potential biomarker of the defect called MSI (Russo et al., 2019). Previous studies have shown that MMR deficiency and MSI are highly sensitive predictors of multiple types of cancers, and MSI is also useful to determine the response and sensitivity of immunotherapy (Dudley et al., 2016; Hause et al., 2016; Baretti and Le, 2018). TMB refers to the measurement of the complete number of mutations in cancer cells, which is also a novel therapeutic target (Chan et al., 2019). Also, it appears as an effective method to predict the prognosis of patients and respond to ICIs for cancer treatment (Yarchoan et al., 2017; Chan et al., 2019; Samstein et al., 2019). DNA methylation, an epigenetic mechanism, is another novel predictor for tumorigenesis. According to this study results, we found that FURIN expression was highly related to 5 MMR genes in 14 cancers and to MSI in 11 cancers. In addition, they implied that FURIN expression was strongly correlated with TMB in 8 cancers and with 4 DNA methyltransferases in 10 cancers. They also showed that the aberrant FURIN expression across cancers may serve as a key factor in the development and progression of cancer for the first time, which may be strongly related to MMR, MSI, TMB, and DNA methylation.

Currently, TME has been a hot and trending spot in tumor research. Immune cells are vital stromal compartments in TME, which include neutrophils, natural killer cells, macrophages, dendritic cells, B cells, and T cells. The roles of immune cells are different due to the expression of some key regulators, including microRNAs (Xu et al., 2019). Generally speaking, immune cells display strong antitumor properties. Many studies indicate that immune cells in TME may serve as a crucial factor in the development and progression of multiple cancers (Rabinovich et al., 2007; Beatty and Gladney, 2015; Osipov et al., 2019). However, few studies reveal the role that furin plays in TME. According to this study results, FURIN expression was highly related to six infiltrating immune cells in COAD, KIRC, KIRP, LGG, LUAD, and PCPG. We also used the ImmuneScore and StromalScore to assess the population of the infiltrating immune cells in TME (Galon et al., 2013). This study results indicated that FURIN expression was strongly related to ImmuneScore and StromalScore in 10 types of cancers, respectively. Furthermore, we found the co-expression of FURIN with 47 immune checkpoint markers across cancers, specifically in LGG, GBM, and THYM. The novel results of this study implied that FURIN may recruit and regulate infiltrating immune cells to inhibit or promote the progression of cancers, which strongly reveals that FURIN serves as a key factor in cancer immunity.

Based on previous studies, furin can be a useful indicator among multiple diseases, which is clinically important. Due to the COVID-19, it was found to be a potential therapeutic target and furin inhibitor can suppress virus production (Cheng et al., 2020; Wu et al., 2020). It also plays a critical role in the diagnosis and treatment of cancer. As a predictor, furin has been contained by the predictive model in many cancers, such as cervical cancer (Ju et al., 2020). As for the treatment, the intracellular self-assembly of olsalazine nanoparticles mediated by furin can be used to treat patients with cancer (Yuan et al., 2019). In addition, the molecular self-assembly instructed by furin can activate apoptosis, which is a novel way in cancer therapies (Fu et al., 2020). In this study, we have displayed the expression landscape of FURIN in human pan-cancer, indicated for the first time that FURIN may serve as an important predictive factor in tumor immunity, and revealed the associations between furin and immune therapy. However, this study has presented some shortcomings: first, multiple information from diverse databases were collected for the analysis, which may lead to systematic bias. More detailed and specific studies are needed in the future to solve this problem. Second, this study mainly focused on using bioinformatic tools to analyze FURIN expression and prognosis in human pan-cancer. Although it may be hard to perform *in vivo/in vitro* experiments simultaneously, it will be more convincing to carry out experiments and even the clinical research. Future studies that aim to explore the exact mechanism of FURIN, at cellular or molecular levels, can be carried out based on the bioinformatic results of this study. Another limitation of this study is that the exact mechanism of the relationships between FURIN and tumor immunity and prognosis of patients are still unclear. Future studies that focus on FURIN expression and tumor immunity in specific cancers may present a more convincing viewpoint according to this issue. Nevertheless, this study results delivered meaningful insights into the immunological and prognostic features of FURIN in human pan-cancer. Future studies can benefit from this study and pay more attention to the exact mechanisms that furin impacts the survival outcomes and immunology in multiple cancers.

## Conclusion

To conclude, the present study has revealed that FURIN is correlated with the prognosis of patients and immune infiltration across cancers, specifically in GBM, LGG, and LUAD. Furthermore, FURIN expression was found to be associated with MMR, MSI, TMB, and DNA methylation in multiple cancers. In addition, FURIN expression was strongly associated with the expression of ICIs in various cancers, particularly in LGG, GBM, and THYM. Therefore, FURIN may play a key role as a prognostic biomarker across cancer. This study results may provide a novel and effective immunological antitumor strategy for research of tumor immunity.

## Data Availability Statement

Publicly available datasets were analyzed in this study. This data can be found here: CCLE data was downloaded from the GEO database (https://www.ncbi.nlm.nih.gov/gds/) under the accession number(s) GSE36133. GTEx RNA-seq data was downloaded from the GTEx database (https://gtexport.org/home/) under the accession number(s) ALL-FPKM. TCGA Pan-cancer RNA-seq data was downloaded from the TCGA database (https://gdc.cancer.gov/) under the accession number(s) ALL-FPKM.

## Author Contributions

BZ and SG designed and supervised the study. BZ analyzed the data and wrote the original draft. SG edited the draft. Both the authors have read and approved the final manuscript.

## Conflict of Interest

The authors declare that the research was conducted in the absence of any commercial or financial relationships that could be construed as a potential conflict of interest.

## Footnotes

1 https://gtexport.org/home/

2 https://portals.broadinstitute.org/ccle/

3 https://cistrome.shinyapps.io/timer/

## Nomenclature

MMR: mismatch repair

MSI: microsatellite instability

TMB: tumor mutational burden

ICIs: immune checkpoint inhibitor

OS: overall survival

DSS: disease-specific survival

PFI: progression-free interval

DFI: disease-free interval

PCs: proprotein convertases

IGF1R: insulin-like growth factor 1 receptor

TME: tumor microenvironment

TAMs: tumor-associated macrophages

GTEx: Genotype-Tissue Expression

CCLE: Cancer Cell Line Encyclopedia

TCGA: The Cancer Genome Atlas

TIMER: Tumor Immune Evaluation Resource

BRCA: bladder urothelial carcinoma

ESCA: esophageal carcinoma

HNSC: head and neck squamous cell carcinoma

LUAD: lung adenocarcinoma

STAD: stomach adenocarcinoma

UCEC: uterine corpus endometrial carcinoma

CHOL: cholangiocarcinoma

LUSC: lung squamous cell carcinoma

READ: rectum adenocarcinoma

ACC: adrenocortical carcinoma

KIRC: kidney renal clear cell carcinoma

KIRP: kidney renal papillary cell carcinoma

LGG: lower grade glioma

MESO: mesothelioma

OV: ovarian serous cystadenocarcinoma

PCPG: pheochromocytoma and paraganglioma

PRAD: prostate adenocarcinoma

TGCT: tenosynovial giant cell tumor

THYM: thymoma

THCA: thyroid carcinoma

UVM: uveal melanoma

COAD: colon adenocarcinoma

PAAD: pancreatic adenocarcinoma

SCRC: sporadic colorectal cancer

SARC: sarcoma

DLBC: diffuse large B-cell lymphoma

SKCM: skin cutaneous melanoma

BLCA: bladder urothelial carcinoma

Tregs: regulatory T cells

TIDCs: tumor-infiltrated dendritic cells

MHC: major histocompatibility complex
